# Multimodal Surgical and Medical Treatment for Extensive Rhinocerebral Mucormycosis in an Elderly Diabetic Patient: A Case Report and Literature Review

**DOI:** 10.1155/2014/527062

**Published:** 2014-05-27

**Authors:** Paola Di Carlo, Roberto Pirrello, Giuliana Guadagnino, Pierina Richiusa, Antonio Lo Casto, Caterina Sarno, Francesco Moschella, Daniela Cabibi

**Affiliations:** ^1^Department of Sciences for Health Promotion and Mother-Child Care “G. D'Alessandro”, University of Palermo, Via del Vespro 127, 90127 Palermo, Italy; ^2^Plastic and Reconstructive Surgery, Department of Surgical, Oncological and Stomatological Sciences, University of Palermo, Via del Vespro 129, 90127 Palermo, Italy; ^3^Laboratory of Molecular Endocrinology, Section of Endocrinology, Biomedical Department of Internal and Specialized Medicine (Di.Bi.M.I.S), University of Palermo, Via del Vespro 127, 90127 Palermo, Italy; ^4^Section of Radiology, Di.Bi.Me.F., University of Palermo, Via del Vespro 129, 90127 Palermo, Italy; ^5^Section of Radiology, “P. Giaccone” Teaching Hospital Palermo, Via del Vespro 127, 90127 Palermo, Italy

## Abstract

Diabetes is a well-known risk factor for invasive mucormycosis with rhinocerebral involvement. Acute necrosis of the maxilla is seldom seen and extensive facial bone involvement is rare in patients with rhino-orbital-cerebral mucormycosis. An aggressive surgical approach combined with antifungal therapy is usually necessary. In this report, we describe the successful, personalized medical and surgical management of extensive periorbital mucormycosis in an elderly diabetic, HIV-negative woman. Mono- or combination therapy with liposomal amphotericin B (L-AmB) and posaconazole (PSO) and withheld debridement is discussed. The role of aesthetic plastic surgery to preserve the patient's physical appearance is also reported. Any diabetic patient with sinonasal disease, regardless of their degree of metabolic control, is a candidate for prompt evaluation to rule out mucormycosis. Therapeutic and surgical strategies and adjunctive treatments are essential for successful disease management. These interventions may include combination therapy. Finally, a judicious multimodal treatment approach can improve appearance and optimize outcome in elderly patients.

## 1. Introductions

Mucormycosis is an emerging angioinvasive infection caused by the ubiquitous filamentous fungi belonging to the Mucorales order [1]. It is the third most common invasive fungal infection after candidiasis and aspergillosis and it is a devastating disease.

There has been a significant increase in incidence over the last two decades due to predisposing factors such as uncontrolled diabetes mellitus with or without ketoacidosis, corticosteroid treatment, organ transplants, allogenic stem cell transplants, neutropenia, trauma and burns, malignant hematologic disorders, and deferoxamine therapy in patients receiving hemodialysis [2, 3]. Diabetes was the most common risk factor in several case series involving immunocompetent patients [3, 4]. Mucormycosis can occur in six different forms: rhinocerebral, pulmonary, cutaneous, gastrointestinal, disseminated, and uncommon presentation; the most frequent sites of infection are pulmonary, rhinocerebral, cutaneous, and disseminated [2]. This fungal infection is characterized by a rapid progression to disseminated infection and a high mortality rate, which is why early diagnosis and timely intervention lead to a better outcome [2, 4, 5]. Although considerable progress has been made using a multimodal approach to treating invasive mucormycosis, a number of clinical issues still need to be resolved and therapeutic outcome remains far below our expectations in many specific areas.

Recently, ESCMID and ECMM joint clinical guidelines pointed out that no clinical trials have been conducted to evaluate the timing of fever-driven treatment against mucormycosis [5]. Different studies in murine models and in vitro have explored the efficacy of combination therapy against members of the Mucorales order [6, 7].

Currently, novel regimens for the treatment of mucormycosis include a combination of lipid-based amphotericin plus either an echinocandin or itraconazole or both [7–9]. Although encouraging, recent guidelines stipulate that there is still a lack of sufficient data to support a recommendation for combination therapy as the first-line treatment for mucormycosis [5, 10].

In this report, we describe the successful, personalized medical and surgical treatment of extensive periorbital mucormycosis complicated by internal carotid thrombosis in an elderly woman. Monotherapy with liposomal amphotericin B (L-AmB) or posaconazole (PSO) and combination of antifungal therapeutic strategies are discussed.

## 2. Case Presentation

In May 2013, a 72-year-old HIV-negative woman of Italian origin and nationality was admitted to the plastic surgery unit of the “P. Giaccone” Teaching Hospital in Palermo, Italy, with extensive necrosis on the right side of her face.

The patient had a past medical history of hypertension, chronic obstructive pulmonary disease (COPD), type 2 diabetes mellitus (DM), and a recent amaurosis fugax episode.

Home medications included atorvastatin 80 mg daily, furosemide 40 mg daily, antiplatelet therapy with aspirin (325 mg daily), metformin 500 mg twice/day, and irregular treatment with fluticasone propionate plus salmeterol by nebulizer.

The disorder started with symptoms consistent with sinusitis; sinus pain, drainage, and soft tissue swelling were reported over a period of 7 days.

When she was admitted to the Emergency Department of the Paolo Giaccone University Hospital, she had been suffering from severe headaches, facial numbness, and blurry vision for 20 hours. Vital signs on admission were as follows: temperature 36.5°C, blood pressure 140/75 mmHg, pulse 95 beats/min, respiratory rate 26 breaths/min, and oxygen saturation 92% on room air.

Relevant laboratory values on admission were as follows: leucocytosis with 14.600 leucocytes with prevalence of segmented elements (83%), glucose 220 mg/dL, blood urea nitrogen 76 mg/dL, creatinine 2 mg/dL, and creatinine clearance (CrCl) 30 mL/min.

**Figure 1:**
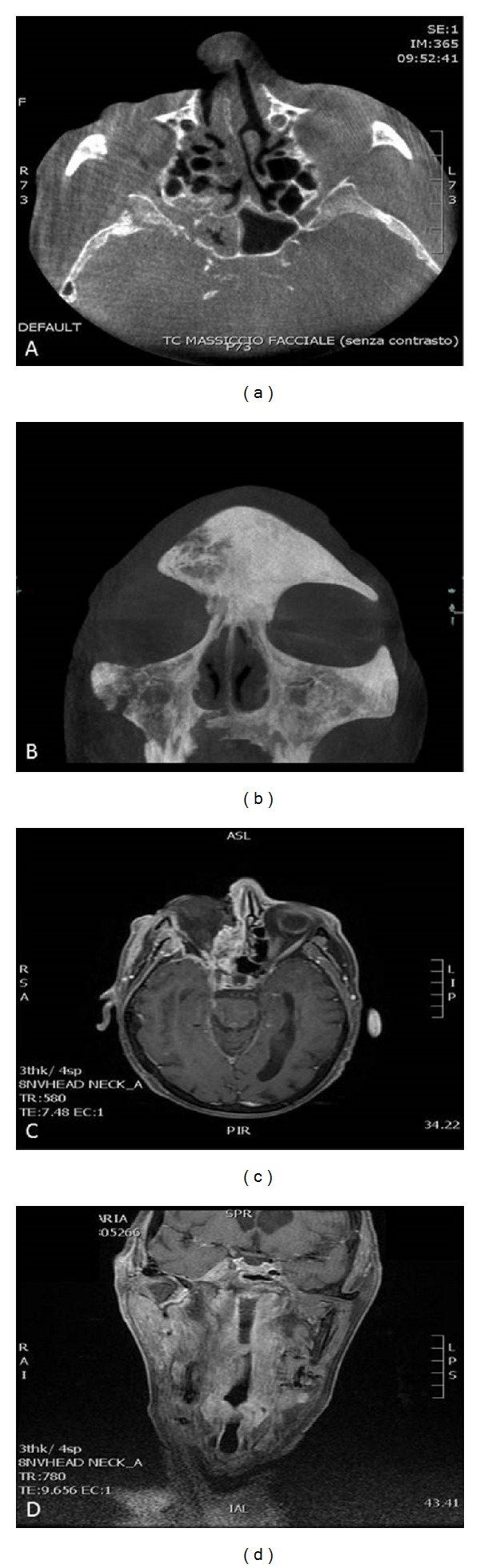
Base (axial plane) CT image shows the presence of a soft tissue density mass in the right sphenoid sinus, occluding the sinus lumen and the spheno-ethmoid osteum also osteostructural alterations, mainly lytic, of the lateral sinus wall and in part of the large sphenoid wing (a); base (coronal plane) CT image with MIP (maximum intensity project) reconstruction shows lytic osteostructural alteration of the right side of the hard palate, with interruption of the cortical contour and, in part, of the alveolar process of the jaw in the first sextant (b); axial T1-weighted MRI image after mdc administration shows meningeal enhancement in the right temporal lobe. Also enhancement of the temporiun on the right side and the Sylvian aqueduct indicating transcompartment fungal infection involvement (c); coronal T1-weighted MRI image after administration of mdc shows a large amount of phlogistic tissue, intensely and homogenously enhanced after mdc, which fills the Meckel's cavity and engulfs the intracavernous branches of the internal carotid artery in the absence of perceptible ischaemic lesions of the brain parenchyma (d).

CT images (Figures 1(a) and 1(b)) showed spreading cellulitis on the right side of the face and cervicofacial areas with loss of nasobuccal substance and lytic-type osteostructural alteration on the right side of the hard palate with interruption of the cortical contour and, in part, of the alveolar process of the jaw in the first sextant; magnetic resonance imaging (Figures 1(c) and 1(d)) showed meningeal involvement and localizations in the cerebral parenchyma and aqueduct of Sylvius; a large amount of phlogistic tissue fills the Meckel's cavity and engulfs the intracavernous branches of the internal carotid artery.

**Figure 2:**
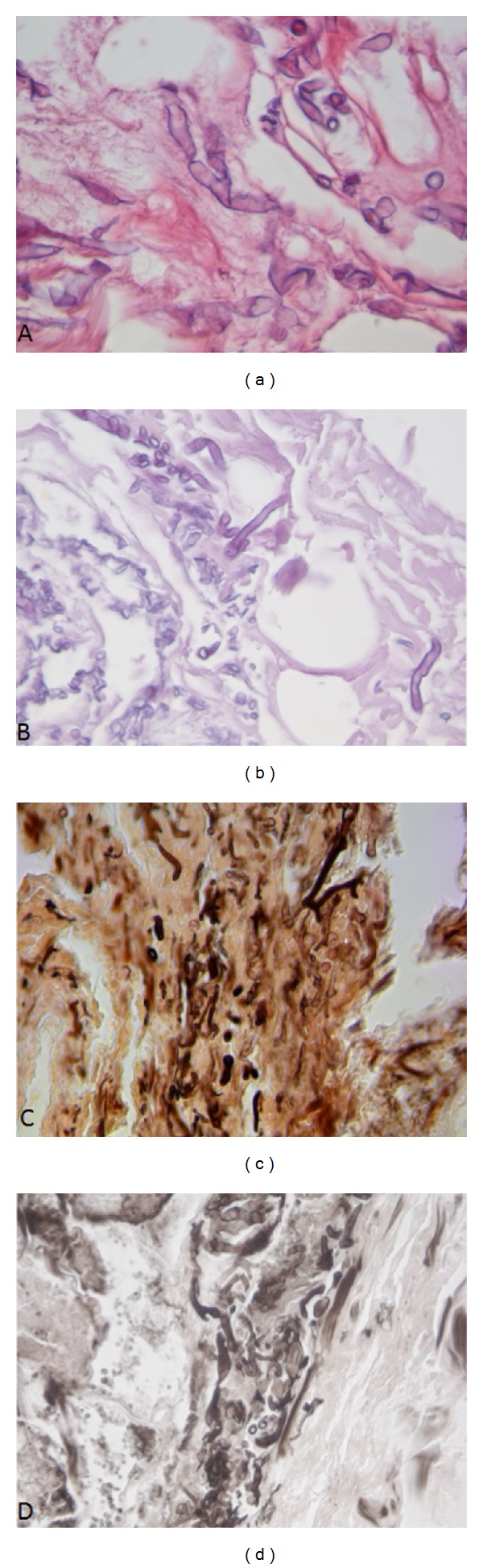
Spores and broad, stubby, irregularly-shaped hyphae, in many cases with nondichotomous right-angle branching; (a) hematoxylin-eosin staining, (b) PAS staining, and (c)-(d) Gomori silver methenamine staining; original magnification = 400x.

The patient underwent nasal endoscopy and a biopsy was performed to remove tissue from the sites of infection for examination. Histopathology tests showed fragments of necrotic connective tissue associated with spores and stubby and broad hyphae. They were irregularly shaped, in many cases, with nondichotomous right-angle branching. They were more evident by hematoxylin-eosin staining (H-E) Figure 2(a) than by PAS Figure 2(b). Gomori silver methenamine (GMS) staining was also positive (Figures 2(c) and 2(d)). These findings were in keeping with the diagnosis of invasive mucormycosis.

Culture of a small fragment of a biopsy specimen onto Sabouraud dextrose agar showed vigorous growth of colonies that were consistent with “Mucorales” on the basis of both gross colony morphology (floccose or “woolly”) and microscopic features of slide culture (aseptate hyaline hyphae and sporangium morphology: globose and deliquescent). The isolate was not speciated and molecular identification was not performed.

Antimicrobial susceptibility testing (AST) was performed using broth microdilution as described in the NCCLS M38-A document [11] and as previously reported [12].

Amphotericin B (AMB) showed a MIC < 1 *μ*g/mL.

On the second day of hospitalization, the woman's clinical condition precluded surgical debridement of infected tissues (exacerbations of COPD) and hyperbaric oxygen (catarrhal otitis media); therefore monotherapy with parenteral liposomal amphotericin B (L-AmB) at a dose of 3 mg/kg/day was started.

**Figure 3:**
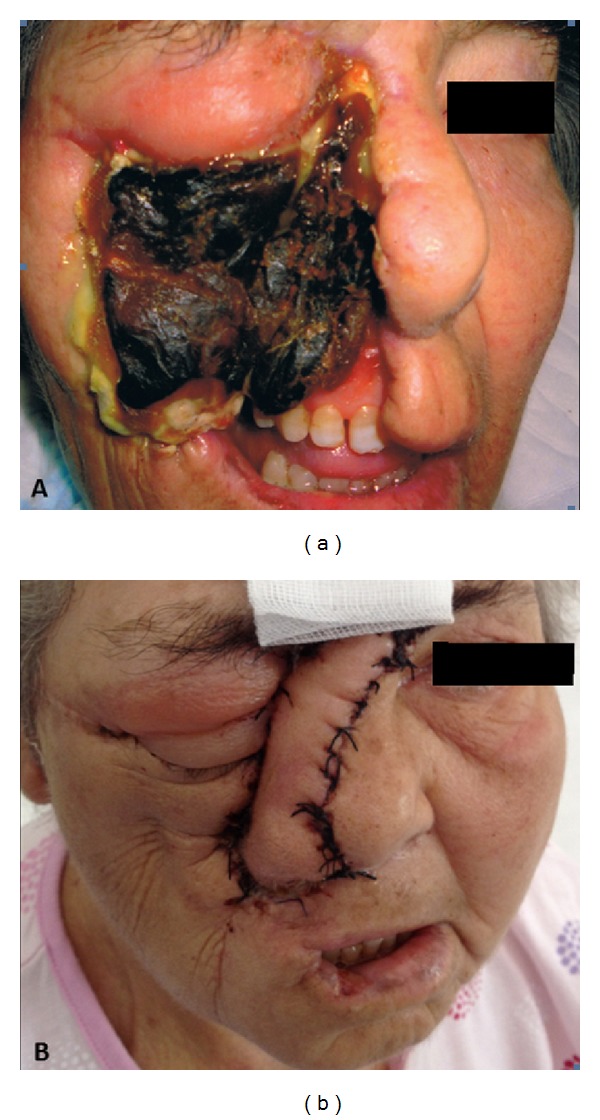
Patient on admission (a) and after treatment (b).

Figure 3(a) shows the patient's lesions in the first preoperative period.

No improvement was observed after 3 weeks of antifungal monotherapy; therefore combination therapy of posaconazole (400 mg twice daily) plus L-AmB at the above-mentioned dosage was started.

During hospitalization, the patient's glucose levels were restored to normal and kept in the normal range by switching from oral medication to injected insulin.

After two weeks of combined antifungal treatment, there was a general improvement in the patient's state of health; therefore an aggressive surgical debridement with orbital exenteration was performed. This was followed by Mustardè flap reconstruction of residual substance in the maxillary zygomatic region and local flap reconstruction on the upper lip.

Two surgical specimens were sent to the pathology laboratory, one measuring about 5 cm from the hemi face and the other measuring 1.5 cm in diameter taken from the right orbital cavity after the eye had been removed. Histological examination showed necrotic tissue with fungal spores and hyphae once again consistent with mucormycosis (data not shown).

However, cultures of surgical specimens were negative.

During hospitalization, the patient refused further surgical treatment, such as a neurosurgical procedure or endovascular intervention.

Combination antifungal therapy was continued in the same dose during the intraoperative phase and postsurgery hospitalization, for a total of 24 days. The patient's renal function, liver function, and blood counts were closely monitored; mean value of CrCl during hospitalization was 33 ± 4 mL/min.

As a result of her optimal response to combined antifungal therapy and good outcome after surgery, the patient was discharged. Considering the extent of the lesions and the patient's underlying condition which precluded hyperbaric oxygen treatment, prolonged combination antifungal therapy was considered essential and an outpatient treatment regimen was prescribed. Nevertheless, despite our concerns for her health, she did not consent to intravenous antifungal treatment with amphotericin B.

Therefore, the patient was discharged to continue monotherapy with posaconazole at home for another 5 weeks. Posaconazole treatment was well tolerated and no adverse effects were detected during the 60 days that the drug was used in combination or in second-line therapy.

After discharge, the patient returned to the plastic surgery clinic to have the surgical wound checked and the dressing changed (Figure 3(b)).

Meanwhile, the patient appeared to be in good health and wound biopsy specimens were negative for yeast infection.

At a recent follow-up examination a year after the discharge, the patient did not show any symptoms of recurrent disease.

## 3. Discussion

Treatment of rhinocerebral mucormycosis requires a multifaceted approach which includes antifungal agents, surgical debridement, and correction of the underlying disease which predisposes the individual to infection.

However, possible long-term complications such as loss of oral and visual function, disfigurement, diminished quality of life, and psychosocial consequences, together with their impact on survival, force us to continue to redefine management strategies for mucormycosis.

There are still a number of controversial issues regarding both surgical and medical interventions.

Although early and extensive surgical debridement appears to enhance survival [13], delayed and less aggressive surgical interventions have also been reported [14].

In the case described here, our medical and surgical approach was influenced by the patient's clinical conditions. In fact, debridement was withheld for 21 days and the patient was given less than the recommended dose of L-AmB [5, 10]. Although high doses of L-AmB (7–10 mg/kg/day) are recommended [5, 10] for invasive mucormycosis and there is no need to reduce the dose in patients with renal disease, we decided to take into account the potential nephrotoxic effects of L-AmB in older patients [15] and we prescribed a dose of 3 mg/kg/daily for the whole course of treatment.

Moreover, our patient showed poor compliance with intravenous medication and she refused further surgical treatment, such as a neurosurgical procedure or endovascular intervention. Although this precluded a multidisciplinary approach, she did however consent to plastic surgery. This decision was perhaps dictated by her fear of isolation and of people's reactions to her appearance. Moreover, the patient was elderly and it was more important for her not to appear as a “monster” than to extend her life.

After discharge, our patient refused intravenous treatment and was therefore prescribed monotherapy with posaconazole. ESCMID and ECMM guidelines recommend posaconazole prophylaxis for mucormycosis and for salvage treatment in patients resistant to other antifungal agents used in previous treatments [5]. In this regard, our patient's favorable outcome was consistent with other published cases in which the drug was frequently and successfully used in combination or second-line therapy [16].

We added posaconazole to the L-AmB treatment regimen before and after the patient's surgical debridement. Single case reports or case series available in the literature have demonstrated the efficacy of a combination of posaconazole plus L-AmB [17, 18]. James J. Mezhir reported that patients with invasive mucormycosis were successfully treated with a combination antifungal therapy of posaconazole plus L-AmB without the need for surgical debridement [19].

Another potential endpoint for clinical studies that needs to be spotlighted is the duration and dosage of antifungal therapy using single-drug or combination strategies. Our patient received antifungal drug medication for about 80 days. Although there are no exact indications regarding the duration of treatment for rhinocerebral mucormycosis, we believe that, in our patient's case, prolonged antifungal therapy was an important factor for survival.

Moreover, our patient took atorvastatin which, like other statins, has shown activity against Mucorales and displayed a synergistic effect when combined with azole [5].

Infected tissue biopsy is of fundamental importance in mucormycosis diagnosis, as it can reveal the distinctive hyphae characteristics (broad, ribbon-like, irregularly-shaped, coenocytic, or sparsely septate, in many cases with nondichotomous and right-angle branching) which are suggestive of disease.

Nevertheless, culture often yields no growth and histopathological identification of an organism which is structurally identical to Mucorales is frequently the only evidence of infection [20].

Although our patient's underlying condition precluded hyperbaric oxygen (HOB), it is considered a potentially significant adjunctive treatment for invasive fungal infections [5, 12, 21]. There is insufficient evidence to determine its usefulness as a standard of care in these infections, but it may be warranted in patients who appear to be deteriorating, in spite of maximal surgical and medical therapy [5, 13].

Despite the emergence of new antifungal agents, mucormycosis has an exceedingly high mortality rate; therefore improving host response to infection appears to be a critical prognostic factor. Our case suggests that advanced age probably contributes to a weaker immune response to filamentous fungi such as zygomycetes due to changes in T cell responses [22]. The precariousness of the situation is accentuated by underlying conditions or comorbidities such as cardiovascular disease or COPD in the elderly population [23].

Finally, as this case report illustrates, medical and surgical treatment of extensive rhinocerebral mucormycosis can be successful [24], but the importance of long-term follow-up and the potential for disease recrudescence must be taken into account.

## 4. Conclusions

In conclusion, two interesting findings emerge from this case report. Firstly, a long- term, multimodal treatment approach consisting in combined antifungal drug therapy (posaconazole + liposomial amphotericin B) and timely surgical debridement can lead to an improvement in appearance and in long-term outcome.

Secondly, our case report highlights the complex nature of invasive fungal disease management in patients affected by multiple concurrent chronic conditions, as is often the case in elderly patients.
